# Raman spectroscopic signatures of carotenoids and polyenes enable label-free visualization of microbial distributions within pink biofilms

**DOI:** 10.1038/s41598-020-64737-3

**Published:** 2020-05-07

**Authors:** Hiroto Horiue, Mai Sasaki, Yuki Yoshikawa, Masanori Toyofuku, Shinsuke Shigeto

**Affiliations:** 1 Department of Chemistry, Graduate School of Science and Technology, Kwansei Gakuin University, 2-1 Gakuen, Sanda Hyogo, 669-1337 Japan; 2 Faculty of Life and Environmental Sciences, University of Tsukuba, Ibaraki, 305-8572 Japan; 3 Microbiology Research Center for Sustainability, University of Tsukuba, Ibaraki, 305-8572 Japan

**Keywords:** Analytical chemistry, Microbiology, Optical techniques

## Abstract

Pink biofilms are multispecies microbial communities that are commonly found in moist household environments. The development of this pink stain is problematic from an aesthetic point of view, but more importantly, it raises hygienic concerns because they may serve as a potential reservoir of opportunistic pathogens. Although there have been several studies of pink biofilms using molecular analysis and confocal laser scanning microscopy, little is known about the spatial distributions of constituent microorganisms within pink biofilms, a crucial factor associated with the characteristics of pink biofilms. Here we show that Raman spectroscopic signatures of intracellular carotenoids and polyenes enable us to visualize pigmented microorganisms within pink biofilms in a label-free manner. We measured space-resolved Raman spectra of a pink biofilm collected from a bathroom, which clearly show resonance Raman bands of carotenoids. Multivariate analysis of the Raman hyperspectral imaging data revealed the presence of typical carotenoids and structurally similar but different polyenes, whose spatial distributions within the pink biofilm were found to be mutually exclusive. Raman measurements on individual microbial cells isolated from the pink biofilm confirmed that these distributions probed by carotenoid/polyene Raman signatures are attributable to different pigmented microorganisms. The present results suggest that Raman microspectroscopy with a focus on microbial pigments such as carotenoids is a powerful nondestructive method for studying multispecies biofilms in various environments.

## Introduction

Over the last three decades, the traditional view that microorganisms exist as planktonic cells floating in a fluid has been found to be true only for certain culture conditions realized in the laboratory environment. It is now widely recognized that in the actual environment, irrespective of whether natural or anthropogenic, microorganisms form and live in communities known as biofilms^1–4^. Biofilms are assemblages of microorganisms embedded in the matrix of self-produced extracellular polymeric substances and grow on many different types of biotic or abiotic surfaces. Because this mode of microbial life is responsible for a variety of microbiological phenomena such as microbial infection^5,6^, biofouling^7^, and bioremediation^8^, considerable efforts have been made to elucidate the properties and functions of biofilms. A majority of earlier biofilm studies focused on model biofilms consisting of single microbial species. Although those studies on single-species biofilms have helped develop a fundamental understanding of what biofilms essentially are, more and more research currently is being directed toward multispecies biofilms, which play a central role in biological, medical, and environmental fields^9–12^.

Within a multispecies biofilm, individual microorganisms occupy a specific microniche, depending not only on physicochemical factors such as oxygen and nutrient availability but also on symbiotic interactions with surrounding microorganisms. A consequence of this is heterogeneous microbial distributions in the biofilm^13^. Characterization of multispecies biofilms in a spatially resolved manner is thus of great importance to fully understand them. Confocal laser scanning microscopy (CLSM) using fluorescent reporter proteins has been widely applied to visualize microorganisms in living biofilms. This technique, however, usually requires the transformation systems for target microorganisms to be already established. In addition, its use is limited to aerobic conditions under which fluorescent proteins can function appropriately. These limitations often make it difficult to apply the standard CLSM technique to complex environmental microbial communities^14^.

Here, we present a new approach based on Raman microspectroscopy to visualizing the distributions of microorganisms within multispecies biofilms without the need of molecular techniques used for CLSM. We utilized characteristic Raman signatures of a specific class of intracellular biomolecules, that is, polyenes including carotenoids. They are commonly found in pigmented microorganisms and serve as an excellent endogenous probe for visualization purposes. The multispecies biofilms investigated in this study are pink biofilms. Pink biofilms are one of the most familiar biofilms that we encounter in everyday life. This nuisance is easily formed by *Methylobacterium* and other microorganisms in moist household environments such as bathrooms (floors^15^, showerheads^16^, shower curtains^17^, drains, etc.) and, once it develops, it becomes quite cumbersome to remove due to high tolerance to both drying and chlorine-containing cleaning agents^18^. Studies have shown that pink biofilms are a potential reservoir of opportunistic pathogens for immunocompromised patients^19,20^. Despite their medical implications, nondestructive characterization of pink biofilms is still scant.

In this work, we carried out two Raman experiments: Raman imaging of a pink biofilm empowered by multivariate curve resolution–alternating least-squares^21,22^ (MCR–ALS) and Raman microspectroscopy of isolates from the same pink biofilm. The first experiment detected three types of Raman spectra that are associated with carotenoids and structurally similar but different polyenes. The detected carotenoids/polyenes exhibited mutually exclusive distributions within the pink biofilm, suggesting that the distributions mapped out by the polyene Raman signatures directly reflect those of different pigmented microorganisms. The second experiment confirmed this hypothesis by looking into the Raman spectra of individual cells of seven major microorganisms isolated from the pink biofilm. Our results demonstrate that the Raman signatures of microbe-produced polyene compounds could be a facile and powerful probe for label-free visualization of microbial distributions within complex multispecies biofilms.

## Results and Discussion

### Raman spectral signatures of pink biofilms

**Figure 1:**
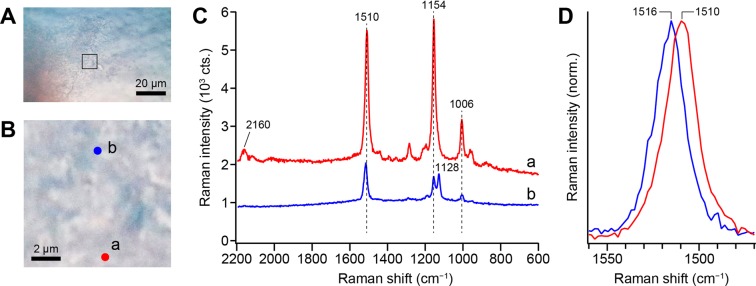
Optical microscopy and Raman microspectroscopy measurements of a pink biofilm collected from the drain of a bathroom. (**A**) Optical micrograph of the pink biofilm. Scale bar, 20 μm. (**B**) Enlarged view of the region in which we performed a Raman imaging experiment. This region is indicated by a square in A. Scale bar, 2 μm. (**C**) 532-nm excited Raman spectra (raw data) measured at points a (red line) and b (blue line) shown in B. The spectra were taken with 1 mW laser power and a 0.5 s exposure time. Neither smoothing nor noise reduction was performed. (**D**) Comparison of the C=C stretching band in the Raman spectra measured at points a (red line) and b (blue line). The bands are normalized to the peak height.

We visually identified and collected pink biofilms grown near drains of household bathrooms. Microscopic images of a representative pink biofilm shown in Fig. 1A,B reveal some textures that reflect biofilm structure, but of course, these optical micrographs provide no molecular information. Figure 1C shows the 532-nm excited Raman spectra of the pink biofilm observed at two representative points a and b designated in Fig. 1B. The confocal Raman microspectrometer that we used here is described in detail in the Methods section. The Raman spectrum at point a (Fig. 1C, red line) is dominated by three sharp, distinct Raman bands peaking at 1006, 1154, and 1510 cm^−1^. This spectral pattern very much resembles what has been observed for *Rhodococcus* sp. SD-74 biofilms in our previous work^23^ and is characteristic of carotenoids with all-*trans* configuration of the conjugated C = C chain. The assignments of the three prominent Raman bands are well established: the 1006 cm^−1^ band is attributed to the C–CH_3_ rocking mode, the 1154 cm^−1^ band the C–C stretching mode (coupled with C–H in-plane bending), and the 1510 cm^−1^ band the C = C stretching mode of the conjugated chain in carotenoids. A weak feature at ~2160 cm^−1^ arises from the combination band of the 1006 and 1154 cm^−1^ bands^24^. The predominance of the carotenoid bands results from the resonance Raman effect. Because the excitation wavelength used in this study (532 nm) is in resonance with the S_0_ → S_2_ (π-π*) electronic transition of carotenoids, carotenoid bands are selectively enhanced by a factor of >10^4^. As a result, nonresonant Raman bands of other biomolecules such as constituents of microbial cells (proteins, DNA/RNA, and phospholipids) and of biofilm architecture (polysaccharides) are buried under overwhelmingly strong carotenoid signatures.

Pink biofilms mainly comprise pigmented microbial cells that may synthesize and accumulate carotenoids, so it comes as no surprise that they exhibit a typical carotenoid Raman spectrum. However, the Raman spectrum observed at point b (Fig. 1C, blue line) shows an additional band at 1128 cm^−1^ in the proximity of the C–C stretching band at 1154 cm^−1^. This is unusual for all-*trans* carotenoids. Besides the presence of this band, we note that (i) the overall Raman intensity is smaller at point b than at point a, (ii) the combination band at 2160 cm^−1^ is absent in the spectrum at point b, and (iii) the peak frequency of the C = C stretching band slightly differs (1510 cm^−1^ for point a *versus* 1516 cm^−1^ for point b; see Fig. 1D).

**Figure 2:**
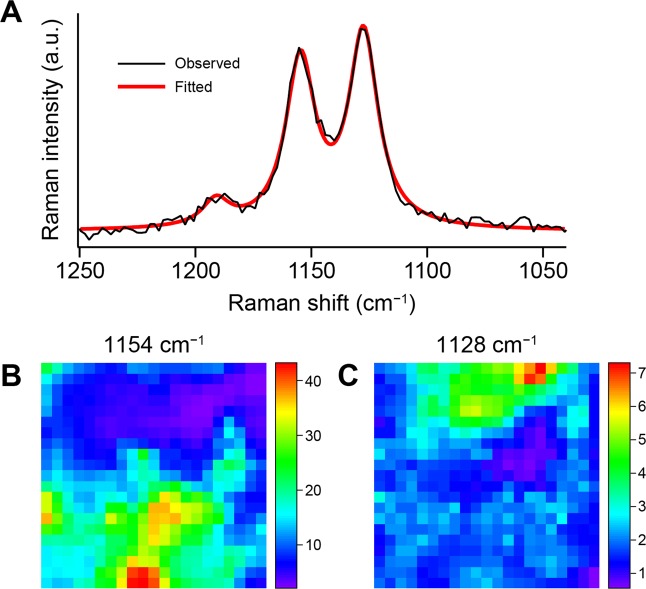
Construction and comparison of the Raman images at 1128 and 1154 cm^−1^. (**A**) Baseline-corrected Raman spectrum at point b in Fig. 1B (black line) and best fit to a sum of three Lorentzian functions (thick red line). The baseline correction was done using polynomial functions (see Methods for details). (**B,C**) Distribution maps of the area intensities of the Lorentzian functions accounting for the 1154 cm^−1^ band (**B**) and the 1128 cm^−1^ band (**C**). The scanned area corresponds to Fig. 1B. The Raman images are displayed in rainbow pesudocolor with red representing the highest intensity and purple the lowest.

To examine whether the 1128 cm^−1^ band is also observed at other locations within the biofilm, we performed a Raman imaging experiment on the 10 μm × 10 μm region shown in Fig. 1B by raster scanning the biofilm sample. As illustrated in Fig. 2A, the baseline-corrected Raman spectrum in the C–C stretching region (1040–1250 cm^−1^) at each point was fit to a sum of three Lorentzian functions (see Methods for details) accounting for the 1128 and 1154 cm^−1^ bands plus a minor band at ~1190 cm^−1^. The area intensities so determined were used to generate Raman intensity distribution maps (i.e., Raman images^25^). The intensity of the 1154 cm^−1^ band is distributed in the lower part of the scanned region (Fig. 2B), whereas that of the 1128 cm^−1^ band is localized in the upper part (Fig. 2C) and shows little overlap with the Raman image at 1154 cm^−1^. This result seems to indicate that the molecular species giving rise to the 1128 and 1154 cm^−1^ band are produced by different microorganisms that occupy different microniches within the pink biofilm.

### MCR–ALS analysis reveals two kinds of polyenes

We then asked whether the 1128 cm^−1^ band is attributable to carotenoids as with the 1154 cm^−1^ band, or other biomolecules. Because it is unlikely that this signature is the only intense Raman-active band for the corresponding molecular species, there should be other bands lying very close to the carotenoid bands. To fully reveal the spectral features of the pink biofilm constituents, we analyzed the Raman hyperspectral imaging data of the pink biofilm using MCR–ALS. We have previously applied this multivariate analysis method to a variety of living cell systems including fission yeast *Schizosaccharomyces pombe* cells^21,26^, human colon cancer cells^22^, and hyphae of the filamentous fungus *Aspergillus nidulans*^27^. MCR–ALS decomposes Raman hyperspectral imaging data into the intrinsic Raman spectra and spatial concentration profiles (Raman images) of major components with non-negativity constraints, assuming that each space-resolved spectrum is approximately expressed as a linear combination of those components. Both spectral intensities and concentrations of each component are inherently non-negative, and this puts a premium on imposing non-negativity constraints, because the decomposition results yielded under those constraints are physically interpretable in a more straightforward manner than those obtained from other commonly used multivariate techniques such as principal component analysis (PCA) and partial least squares-discriminant analysis (PLS-DA).

**Figure 3:**
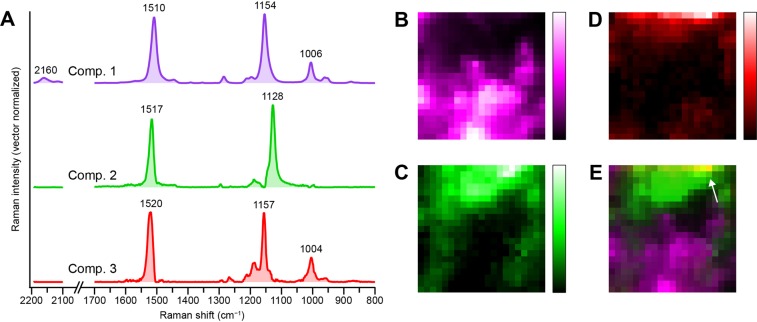
MCR–ALS Analysis of the Raman hyperspectral imaging data of the pink biofilm assuming three components (*k* = 3). (**A**) Intrinsic Raman spectra of the three components (denoted 1–3). Each spectrum has been normalized so that the sum of intensities at all Raman shifts is equal to unity. The spectra are vertically offset for clarity of display. (**B–D**) Raman images of components 1 (**B**), 2 (**C**), and 3 (**D**). The Raman images are shown in pseudocolor. (**E**) Merged image of B–D, indicating mutually exclusive distributions of components 1 (magenta) and 2 (green). Colocalization of components 2 (green) and 3 (red) is manifested as a yellow region (indicated by an arrow).

The intrinsic Raman spectra and Raman images derived from an MCR–ALS analysis of the same data as in Fig. 2 are presented in Fig. 3A,B–D, respectively. Here we assumed the number of underlying components, *k*, to be 3, and these components are denoted 1–3. It is clear from Figs. 1 and 2 that at least two components need to be taken into account, but singular value decomposition (SVD) of the data indicates three or more significant singular components (Supplementary Fig. S1). Thus we also attempted MCR–ALS analysis assuming *k* = 4. The results obtained with *k* = 4 (Supplementary Fig. S2) are less satisfactory than those with *k* = 3 shown in Fig. 3, in the following respects. First, there is a component that possesses Raman bands at both 1128 and 1155 cm^−1^ (component 3′; Supplementary Fig. S2A, red), presumably due to an imperfect resolution. Second, the most minor component among the four (component 4′) judging from the signal-to-noise ratio (SNR) of its spectrum shows unreasonable dips at e.g. ~1160 and ~1515 cm^−1^ (Supplementary Fig. S2A, yellow) and a less localized, scattered distribution pattern (Supplementary Fig. S2E). We thus conclude that the MCR–ALS analysis with *k* = 3 brought about the best decomposition results while minimizing the number of components assumed. Additional support for the three-component model we adopted will be provided later on the basis of Raman spectra of isolates from the pink biofilm. Note, however, that this does not necessarily exclude the existence of other components that we may have failed to separate out because of nearly identical spectra and/or spatial distribution profiles to either one of components 1–3.

The Raman spectrum (Fig. 3A, magenta) and Raman image (Fig. 3B) of component 1 appear almost identical to those shown in Fig. 1C (spectrum a) and Fig. 2B, respectively. This component is therefore assignable to the principal carotenoid species. Likewise, the distribution pattern of the image of component 2 (Fig. 3C) is quite akin to the Raman image at 1128 cm^−1^ (Fig. 2C). Consistent with this correspondence, the intrinsic spectrum of component 2 (Fig. 3A, green) does exhibit a Raman band at 1128 cm^−1^. Owing to our MCR–ALS analysis, the entire Raman spectrum of the chemical species giving rise to the 1128 cm^−1^ band has now been gained, which facilitate interpretation of this species. We can see that the presence of a band at 1517 cm^−1^ and the absence of the combination band at ~2160 cm^−1^ in component 2 agree well with the characteristic features found in spectrum b of Fig. 1C, D (see above). Similar Raman spectra with a band at around 1130 cm^−1^ or even lower have been reported for *Flavobacterium*^28,29^ and yellow to purple marine mollusc shells^30^. The carotenoid-like spectral pattern of component 2 is puzzling when one considers that whereas the peak frequency of 1517 cm^−1^ coincides with the carotenoid C = C stretching mode, that of 1128 cm^−1^ is too low for the typical carotenoid C–C stretching mode.

Carotenoid molecules can occur as different isomers with respect to the conjugated C = C chain, so it would be natural to attribute this finding to *cis* isomers of carotenoids. The resonance Raman spectra of mono- (7-, 9-, 13-, and 15-*cis*) and di-*cis* (9,13- and 9,13′-*cis*) isomers of *β*-carotene reported by Koyama and co-workers^31,32^ reveal that the spectral pattern in the C–C stretching region is highly sensitive to the *cis*–*trans* isomerism. However, they all tend to lack an intense Raman band at as low as 1128 cm^−1^ and to display a more complicated C–C stretching pattern with multiple peaks than the spectrum of component 2 does (Fig. 3A, green). Hence, component 2 is unlikely due to a *cis* isomer of carotenoids.

Although it turned out that *cis* isomers of carotenoids cannot well account for component 2, the sensitivity of the C–C stretching profile to *cis* and *trans* forms of *β*-carotene described above led us to further pursue the idea that structural variation involving the conjugated C = C chain may be a key clue to assign component 2. Along this line, we found in the literature that for a carotenoid molecule without the methyl group attached to the conjugated chain, namely, tetradesmethyl-*β*-carotene (19,19′,20,20′-tetranor-*β*,*β*-carotene), the C–C stretching band appears at ~1130 cm^−1^ and not at ~1155 cm^−1^ while the other Raman spectral pattern of carotenoids being almost preserved^33,34^. Desmethyl carotenoids are essentially a specific class of polyene compounds, and we attribute component 2 to such polyenes.

This assignment is consistent with another plausible candidate for component 2, flexirubins. As depicted in Supplementary Fig. S3, flexirubins are polyenes containing a polyenoic acid chromophore and have been reported to show a Raman spectrum very similar to that of carotenoids, with bands at 1004, 1133, 1154, and 1529 cm^−1^ (refs. ^28,29^).

Our MCR–ALS analysis revealed one more component (i.e., component 3) that was not clearly seen from the space-resolved Raman spectra shown in Fig. 1C. This component shows a typical carotenoid Raman spectrum (Fig. 3A, red), just as component 1. However, the C–C and C = C stretching bands of component 3 are observed, respectively, at 1157 and 1520 cm^−1^, significantly upshifted relative to those of component 1 (1154 and 1510 cm^−1^). This result indicates that the carotenoid species responsible for component 3 has a different conjugated chain length from component 1 (see below). In contrast to components 1 and 2, the Raman image of component 3 (Fig. 3D) represents a localized distribution near the upper edge of the mapped region and overlaps in part with the Raman image of component 1 (see a merged image of all three components shown in Fig. 3E). It can be hypothesized from these distinctly different distribution patterns that the Raman images of carotenoid/polyene components 1–3 visualize *microbial heterogeneity* within the pink biofilm.

### Raman measurements on isolates from pink biofilms

**Figure 4:**
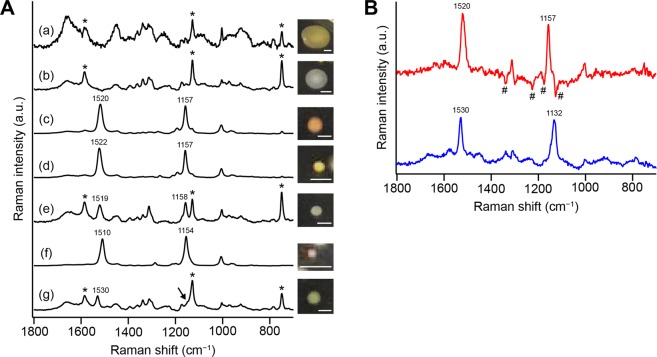
Raman spectra of microbial cells isolated from the pink biofilm sample. (**A**) Averaged Raman spectra (a–g) of 10 different single cells taken from the seven colonies isolated from the same pink biofilm as in Figs. 1–3. Also shown are pictures of the colonies on agar plates (scale bar, 0.5 mm). The Raman spectra were measured in R2A medium. Asterisks denote resonance Raman bands of cytochromes. The arrow highlights a shoulder adjacent to the intense cytochrome band at ~1128 cm^−1^. (**B**) Difference spectra between b and e (e minus b; red line) and between b and g (g minus b; blue line). In doing subtraction, an appropriate coefficient was multiplied to spectrum b so that the major Raman bands of proteins including cytochromes were mostly cancelled. Yet, subtraction artifacts due to inadequate cancelation of the protein Raman bands (marked with #) are seen in the difference spectrum between b and e (red line).

To test this working hypothesis, we measured Raman spectra of isolates from the pink biofilm (same sample as above). We were able to isolate a number of colonies and grouped them into seven (denoted a–g) based on the color, shape, and size of the colony. The Raman spectra of microorganisms a–g, which are averages of ten different cells suspended in liquid medium (see Methods), are shown in Fig. 4A, together with pictures of the colonies.

Spectra a and b do not exhibit carotenoid Raman bands; instead, Raman bands of proteins (plus DNA/RNA)^21,23,25,35^ dominate. Sharp Raman bands at ~748, ~1128, and ~1585 cm^−1^ (indicated by asterisks in Fig. 4A) arise from cytochromes *b* and *c*, which undergo resonance enhancement of Raman intensity upon 532 nm excitation as in carotenoids. These protein/DNA/RNA-dominated spectral patterns are typical of 532 nm-excited Raman spectra of living cells^27,36,37^.

In contrast, spectra c and d both exhibit carotenoid Raman bands whose peak frequencies coincide well with those of MCR–ALS component 3 (Fig. 3A, red). Even though Raman intensities of carotenoids and cytochromes are both enhanced due to the resonance Raman effect, only carotenoid bands are evident in spectra c and d. These pronounced carotenoid intensities might indicate much higher abundance of carotenoids than cytochromes.

Spectrum e is an admixture of the carotenoid bands and the features observed in spectra a and b. We subtracted weighted spectrum b from spectrum e, leaving a “pure” carotenoid spectrum (Fig. 4B, red line) with Raman bands at 1157 and 1520 cm^−1^. The weighting coefficient was determined so that the major Raman bands of proteins including cytochromes were cancelled. Spectrum f is also associated with carotenoids, and its pattern matches the Raman spectrum of component 1 (Fig. 3A, magenta).

Spectrum g resembles spectra b and e at first glance, but they actually represent a couple of differences. There is a prominent Raman band at 1530 cm^−1^ in spectrum g, which is absent in spectrum b. In addition, a Raman band at ~1158 cm^−1^, which is observed in spectrum e, is apparently missing in spectrum g. However, if examined closely, a shoulder can be seen at the higher-frequency side of cytochrome’s 1128 cm^−1^ band (see the arrow in Fig. 4A). To extract the cytochrome-free Raman spectral pattern, we calculated a difference spectrum by subtracting weighted spectrum b from spectrum g, as in the case of spectrum e. The difference spectrum so obtained (Fig. 4B, blue line) unequivocally shows the 1530 cm^−1^ band as expected, and moreover a Raman band at 1132 cm^−1^. The above findings altogether confirm our working hypothesis that carotenoid/polyene components 1–3 derived from the MCR–ALS can indeed be associated with different microorganisms.

### Structural insight into pink biofilm pigments

The results of the Raman measurements on the pink biofilm and on the isolated microorganisms are summarized in Table 1. On the basis of primarily the C–C and C = C stretching frequencies, the pigments detected in the present study can be broadly divided into three groups (denoted I–III). It is well-known that in carotenoids the C = C stretching frequency *ν*(C = C) correlates with the absorption maximum frequency *ν*_max_ = 1/*λ*_max_ (Rimai’s correlation^38^): the larger *ν*_max_, the larger *ν*(C = C). Because there is also a correlation between *ν*_max_ and the number of conjugated C = C double bonds, *N*, *ν*(C = C) can be used to estimate *N* from the observed Raman spectrum. By referring to *ν*(C = C) *versus* 1/*N* correlations reported in previous papers^30,39^, we estimate the effective conjugation length *N* to be 12, 11, and 10 for carotenoids/polyenes of groups I, II, and III, respectively (cf. *N* = 11 for *β*-carotene and 13 for spirilloxanthin). A caveat is that the C = C stretching frequency could be profoundly affected by the surrounding environment of the carotenoid (e.g., solvents and binding proteins^38,40^). This might be a reason for the considerable difference in *ν*(C = C) between the pink biofilm (1517 cm^−1^) and isolated cells (1530 cm^−1^) for group II. Nevertheless, the C = C stretching frequency is useful for eliciting *in situ* information about the polyene structure in pink biofilms as well as in isolated microbial cells.

**Table 1:** Classification of the carotenoids and polyenes detected in the present study.

Carotenoid/polyene group	I	II	III
C–C and C=C stretching frequencies in biofilm (cm^−1^)	1154, 1510	1128, 1517	1157, 1520
C–C and C=C stretching frequencies in isolated cells (cm^−1^)	1154, 1510	1132, 1530	1157, 1520
Estimated C=C double bond number *N*	12	11	10
Isolated microorganisms (labels used in Fig. 4A)	f	g	c, d, e

### Other pink biofilm samples

**Figure 5:**
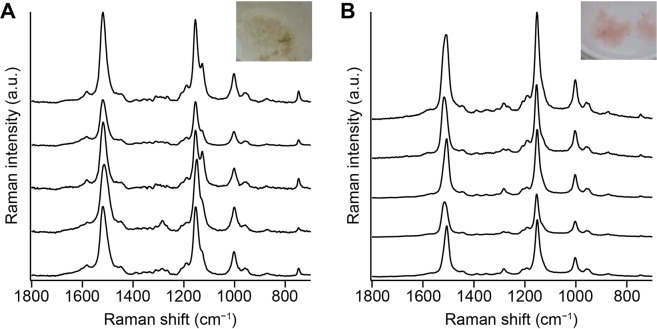
Representative space-resolved Raman spectra measured within pink biofilms collected from a bathroom in different homes in Takarazuka (**A**) and Toyonaka (**B**). These data were recorded with a commercial upright-type Raman microscope (HORIBA XploRA Nano) equipped with a 532-nm laser. The excitation laser power at the sample point was ~4 mW, and the exposure time was 5 s. A 100×, oil-immersion objective (NA = 0.9) was used.

Finally, we asked whether the above results are specific to the bathroom from which the pink biofilm was sampled. We measured space-resolved Raman spectra of two more samples of pink biofilms collected from different bathrooms (Fig. 5). One of them is similar in color to the pink biofilm shown in Fig. 1, whereas the other is more pinkish. In both cases, all representative Raman spectra exhibit carotenoid Raman bands. Most of the Raman spectra displayed in Fig. 5A show a clear doublet in the C–C stretching region consisting of peaks at 1127 and 1155 cm^−1^. In contrast, the 1127 cm^−1^ band is hardly seen in the spectra shown in Fig. 5B. From these results, we can say that although carotenoids are detected within pink biofilms grown in bathrooms irrespective of the environment, whether polyenes similar to component 2 are found or not seem to be dependent on the environment and microbiota.

## Conclusions

Using the Raman signatures of polyenes including carotenoids produced by microorganisms, we have achieved label-free visualization of heterogeneous microbial distribution within pink biofilms. Long image-acquisition time is often claimed to be one of the major shortcomings of the Raman imaging technique, but intense Raman signals of those compounds due to the resonance enhancement can alleviate this problem. In the present case, only 0.5 s exposure time per pixel was sufficient to obtain high-quality Raman spectra (see, for example, Fig. 1C). Methods that enable visualization of the community structure of mixed-microbial biofilms without any pretreatment is highly limited and our Raman method utilizing carotenoid/polyene signatures reveal *in situ* how the pigmented microorganisms occupy distinct microniches within such biofilms. These Raman spectroscopic signatures will potentially be applicable to rapid, on-site detection of specific microbial species (e.g., opportunistic pathogens) in pink biofilms by means of a handheld Raman spectrometer.

Carotenoids are produced by many microorganisms and have diverse biological roles including the light-harvesting function in photosynthetic bacteria^41^. Of particular importance to pink biofilms is their ability to quench reactive oxygen species including superoxide, hydrogen peroxide, and singlet oxygen due to the long π-conjugated system. Microorganisms that make up pink biofilms are frequently exposed to dryness and nutrient deficiencies, and occasionally to cleaning agents, resulting in high levels of stress. They may be using the antioxidant activity of carotenoids to cope with such stressful environments. This speculation is very likely, but many of the biological functions of microbial carotenoids are not yet fully understood. Raman microspectroscopy and imaging could offer a powerful means to address this critical issue in both label-free and space-resolved manner. Moreover, our method can easily be extended to other polyene molecules like component 2 found in the present work and antibiotics as demonstrated for amphotericin B (ref. ^42^).

## Methods

### Samples

Pink biofilm samples were collected with a microspatula from the drain of a bathroom in homes in Hyogo (Sanda and Takarazuka Cities) and Osaka (Toyonaka City) Prefectures, Japan, in April 2019 and October 2017 and in October 2017, respectively. The permission for obtaining those samples was obtained from the house owners. The samples were gently washed with sterilized tap water and transferred under wet conditions to a glass bottom dish for Raman imaging experiments.

For microbial isolation, the suspension of the pink biofilm sample was diluted with sterilized tap water by a factor of 10^4^. 20 μL of the suspension was then spread onto R2A agar plate and left at room temperature for 1 week for colony isolation. Cells were picked from single colonies with different colors, shapes, and sizes, transferred into R2A broth, and then cultured in a shaking incubator at 120 rpm at 30 °C for 1 day. 2 μL of the medium was transferred to a glass bottom dish and statically cultured at room temperature for another 1 day. After substitution of the old medium with sterilized water, the sample was used for Raman measurements.

### Confocal Raman microspectroscopy and imaging

Raman measurements of pink biofilms and microbial cells isolated from the pink biofilms were performed with a laboratory-built confocal Raman microspectrometer. A schematic diagram of the apparatus is depicted in Supplementary Fig. S4. The output at ~532 nm of a continuous-wave semiconductor laser (Coherent Genesis CX532-2000SLM-CDRH) was used as the Raman excitation light. The laser beam was passed through an optical isolator (Thorlabs IO-3-532-LP) and a spatial filter consisting of a 10× objective and a 25 μm pinhole, and subsequently expanded by a factor of 3.6. A portion (~10%) of the expanded beam was introduced into an inverted microscope (Olympus IX73) and focused onto the sample mounted on a 3-axis piezoelectric stage (Nano Control PKM3L160-100U) by a 100×, NA 1.4, oil-immersion objective (Olympus UPLSAPO100X). The laser power at the sample point was adjusted to be 1 mW. Back-scattered light was collected by the same objective and filtered with three volume Bragg gratings (OptiGrate BragGrate notch filters), which eliminated virtually all Rayleigh scattering with an effective elimination bandwidth as narrow as ~10 cm^−1^ (ref. ^43^). After passing through a 100 μm pinhole for confocal detection, the scattered light was analyzed by an imaging spectrometer (SOL Instruments MS3504i) equipped with a 1200 grooves mm^−1^ grating and detected by a thermoelectrically-cooled charge-coupled device (CCD) detector (Andor DU401A-BV) with 1024 × 127 pixels operating at −65 °C. The spectral resolution of our spectrometer was 4 cm^−1^. In Raman imaging experiments on the pink biofilm, the sample was translated with a 0.5 μm step using the piezoelectric stage so that the sample was raster scanned along *X* and *Y* directions over a 10 μm × 10 μm area (i.e., 21 × 21 points) within the biofilm. The sample translation and spectral acquisition using the CCD detector were synchronously controlled with an in-house program written in LabVIEW (National Instruments). The exposure time used to record each Raman spectrum was 0.5 s for the biofilm sample and 60 s for isolated microbial cells. The spatial resolution of our apparatus was estimated to be 0.47 μm in lateral (*XY*) direction and 2.7 μm in axial (*Z*) direction. All spectroscopic measurements were done at room temperature.

### Data analysis

A total of 441 Raman spectra (range 273–2194 cm^−1^) acquired in the Raman imaging experiment were first corrected for baseline using eighth-order polynomial fitting. The resulting baseline-corrected spectra were combined to form 1024 × 441 matrix **A**. Unlike our previous studies^21–23,25^, noise reduction using SVD was not performed on the matrix **A**, because the SNR of the Raman spectra obtained with 532-nm excitation in this study was sufficiently high.

To construct the intensity distribution map of a Raman band of interest, which we call a univariate Raman image^21,25^, the baseline-corrected Raman spectrum at each position was fit to a sum of three Lorentzian functions

$$f(\tilde{\nu })=\mathop{\sum }\limits_{i=1}^{3}\frac{{A}_{i}{\Gamma }_{i}}{{(\tilde{\nu }-{\tilde{\nu }}_{i})}^{2}+{\Gamma }_{i}^{2}}$$

where $\tilde{\nu }$ is Raman shift, and ${\tilde{\nu }}_{i}$, ${A}_{i}$., and ${\Gamma }_{i}$ are, respectively, the peak position, area, and width of the Raman band *i* (*i* = 1–3). The area intensity, ${A}_{i}$, derived from the fitting was then used to generate the univariate Raman image.

Furthermore, in order to obtain spectral and spatial information on principal chemical components underlying the Raman hyperspectral imaging data **A**, it was analyzed with MCR–ALS. The principle of this method and its application to living cell systems have been described in detail elsewhere^21,44,45^. In MCR–ALS, the non-negative data to be analyzed (matrix **A** in the present case) is assumed to be expressed as a linear combination of several independent spectral components. Under this assumption, the equation **A** = **WH** is solved with the constraint that all matrix elements of **W** and **H** are non-negative^46^, by using ALS fitting^47^. In the present study, **W** is a 1024 × *k* matrix whose columns represent intrinsic spectra of the chemical components assumed, and **H** is a *k* × 441 matrix whose rows represent their spatial concentration profiles. The number of components, *k*, must be input *a priori* by the analyst and was set to be *k* = 3 in the present work by reference to the SVD result (see above and Supplementary Fig. S1). The matrices **W** and **H** were initially generated randomly (using Mersenne twister) and optimized with the ALS method with L1-norm regularization (lasso regression^48^) so that the Frobenius norm $\parallel {\bf{A}}-{\bf{W}}{\bf{H}}{\parallel }^{2}$ is minimized. In practice, the following equations with an L1 penalty term of *α*^2^ = 0.004 were solved iteratively:

$$({{\bf{W}}}^{{\rm{T}}}{\bf{W}}+{\alpha }^{2}{\bf{E}}){\bf{H}}={{\bf{W}}}^{{\rm{T}}}{\bf{A}}$$

$$({\bf{H}}{{\bf{H}}}^{{\rm{T}}}+{\alpha }^{2}{\bf{E}}){\bf{W}}={\bf{H}}{{\bf{A}}}^{{\rm{T}}}$$

where **E** is a *k* × *k* matrix, the elements of which are all unity. The ALS optimization was repeated 10000 times to ensure proper convergence.

SVD and spectral fitting with polynomial and Lorentzian functions were both performed on Igor Pro 7.08 (WaveMetrics), whereas MCR–ALS analysis was carried out using a Python-based software developed specifically for spectral imaging applications^21,44^.

## Supplementary information

Supplementary information.
