# Supplementary material for: Raman spectroscopic signatures of carotenoids and polyenes enable label-free visualization of microbial distributions within pink biofilms
Source: Sci Rep. 2020 May 7;10:7704. doi: 10.1038/s41598-020-64737-3 (PMC7206103; doi:10.1038/s41598-020-64737-3)
Supplement: Supplementary file 1 — Supplementary information. [file 41598_2020_64737_MOESM1_ESM.pdf]

Raman spectroscopic signatures of carotenoids and polyenes enable label-free visualization of microbial distributions within pink biofilms

Hiroto Horiue[1], Mai Sasaki[1], Yuki Yoshikawa[1], Masanori Toyofuku[2,3] & Shinsuke Shigeto[1]

[1]Department of Chemistry, Graduate School of Science and Technology, Kwansei Gakuin University, 2-1 Gakuen, Sanda, Hyogo 669-1337, Japan.

[2]Faculty of Life and Environmental Sciences, University of Tsukuba, Ibaraki 305-8572, Japan.

[3]Microbiology Research Center for Sustainability, University of Tsukuba, Ibaraki 305-8572, Japan.

Correspondence and requests for materials should be addressed to S.S. (email: shigeto@kwansei.ac.jp).

Table of contents

Supplementary Figure S1 . . . . S-2

Supplementary Figure S2 . . . . S-2

Supplementary Figure S3 . . . . S-3

Supplementary Figure S4 . . . . S-3

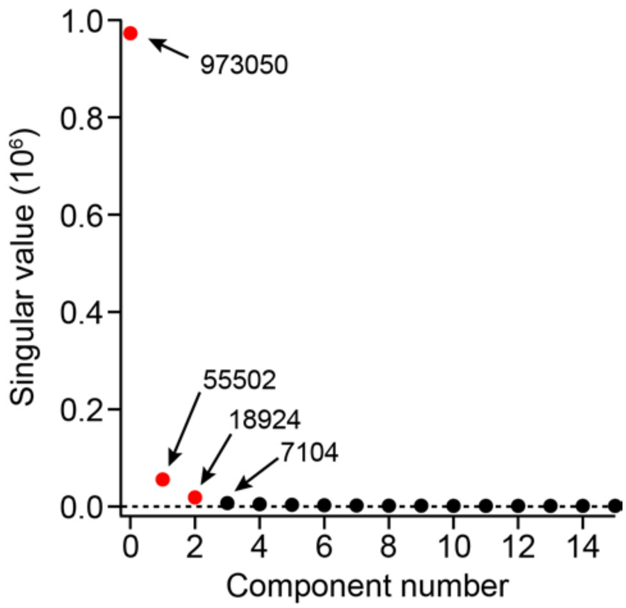

Supplementary Figure S1. Plot of singular values obtained from SVD of the Raman hyperspectral imaging data of the pink biofilm.

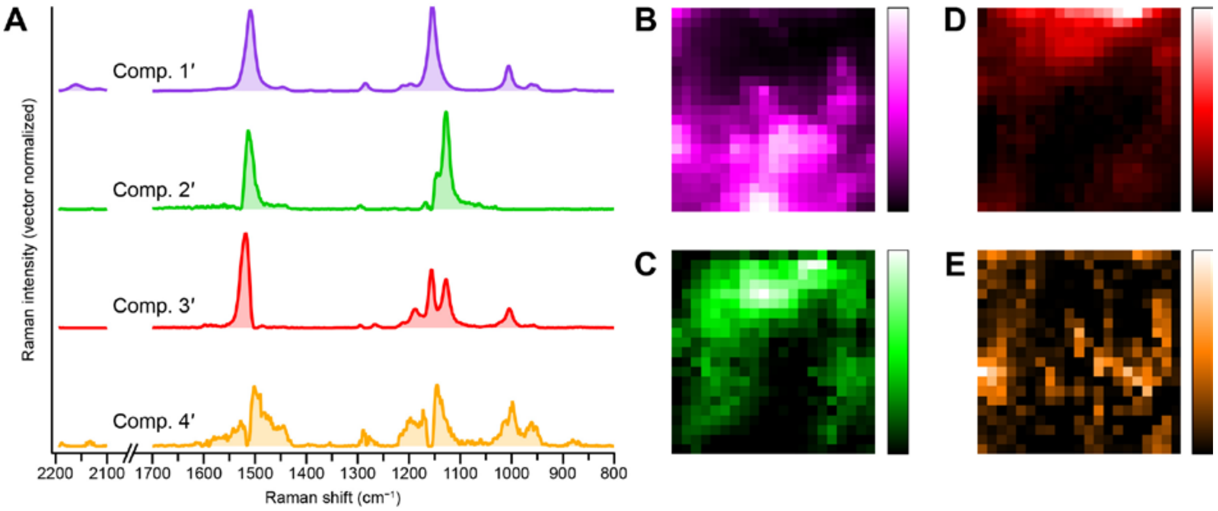

Supplementary Figure S2. MCR–ALS results assuming four components (k = 4). (**A**) Intrinsic Raman spectra of the four components (denoted 1′–4′). Each spectrum has been normalized so that the sum of intensities at all Raman shifts is equal to unity. The spectra are vertically offset for clarity of display. (**B–E**) Raman images of components 1′ (**B**), 2′ (**C**), 3′ (**D**), and 4′ (**E**). The Raman images are shown in pseudocolor.

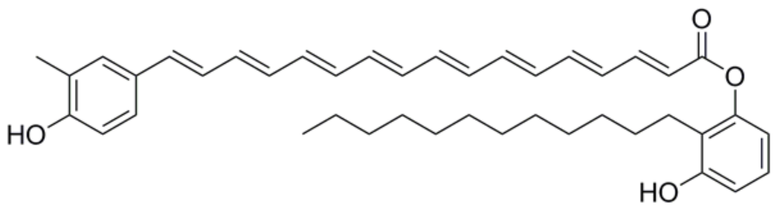

Supplementary Figure S3. Molecular structure of flexirubin.

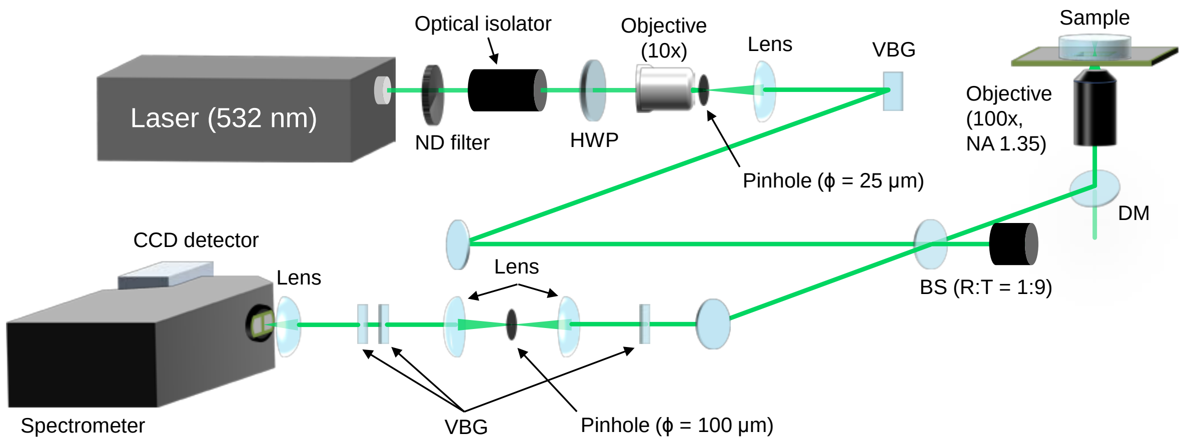

Supplementary Figure S4. Schematic diagram of the laboratory-built, 532 nm-excited confocal Raman microspectrometer. Abbreviations: ND, neutral density; HWP, half-wave plate; VBG, volume Bragg grating; BS, beam splitter; DM, dichroic mirror; CCD, charge-coupled device.
